# Proteo-Transcriptomic Characterization of *Sirex nitobei* (Hymenoptera: Siricidae) Venom

**DOI:** 10.3390/toxins13080562

**Published:** 2021-08-11

**Authors:** Chenglong Gao, Lili Ren, Ming Wang, Zhengtong Wang, Ningning Fu, Huiying Wang, Xiaochen Wang, Tegen Ao, Wensheng Du, Zijin Zheng, Huadong Li, Juan Shi

**Affiliations:** 1 Beijing Key Laboratory for Forest Pest Control, Beijing Forestry University, Beijing 100083, China; gaocl0907@bjfu.edu.cn (C.G.); lily_ren@bjfu.edu.cn (L.R.) mingming66@bjfu.edu.cn (M.W.); wangzhengtong@bjfu.edu.cn (Z.W.); ning_fu@bjfu.edu.cn (N.F.); 2 Sino-France Joint Laboratory for Invasive Forest Pests in Eurasia, INRAE-Beijing Forestry University, Beijing 100083, China; 3 Jilin Forestry Survey and Design Institute, Changchun 130000, China; wanghuiying2677@163.com; 4 Yushu Forest Pest Control and Quarantine Station, Yushu 130400, China; wxc730219@163.com; 5 Tongliao Forest Pest Control and Quarantine Station, Tongliao 028000, China; autogen1@163.com; 6 Heilongjiang Forest Pest Control and Quarantine Station, Haerbin 150000, China; ATang@bjfu.edu.cn; 7 Hegang Green Forest Industry Co., Ltd., Hegang 154100, China; lsgszhz@126.com; 8 Hegang Forestry and Grassland Bureau, Hegang 154100, China; hegangsenbao@163.com

**Keywords:** component, interaction, host, *Pinus*, protein, RNA, toxin, woodwasp

## Abstract

The wood-boring woodwasp *Sirex nitobei* is a native pest in Asia, infecting and weakening the host trees in numerous ecological and commercial coniferous forest plantations. In China, hosts of *S. nitobei* are diverse, so the pest has spread to several provinces of China, resulting in considerable economic and ecological damage. During female oviposition, *S. nitobei* venom along with arthrospores of the symbiotic fungus *Amylostereum areolatum* or *A. chaetica* is injected into host trees, and the combination of these two biological factors causes the death of xylem host trees. The presence of venom alone causes only the yellowing and wilting of needles. In this study, we constructed the venom gland transcriptome of *S. nitobei* for the first time and a total of 15,036 unigenes were acquired. From the unigenes, 11,560 ORFs were identified and 537 encoding protein sequences with signal peptides at the *N*-terminus. Then, we used the venomics approach to characterize the venom composition of female *S. nitobei* and predicted 1095 proteins by liquid chromatography-tandem mass spectrometry (LC-MS/MS) analysis. We focused on seven proteins that were both highly expressed in the venom gland transcriptome and predicted in the crude venom proteome. These seven proteins are laccase-2, laccase-3, a protein belonging to the Kazal family, chitooligosaccharidolytic β-*N*-acetylglucosaminidase, beta-galactosidase, icarapin-like protein, and waprin-Thr1-like protein. Using quantitative real-time PCR (qRT-PCR), we also proved that the genes related to these seven proteins are specifically expressed in the venom glands. Finally, we revealed the functional role of *S. nitobei* venom in the physiological response of host trees. It can not only promote the colonization of symbiotic fungus but contribute to the development of eggs and larvae. This study provides a deeper understanding of the molecular mechanism of the woodwasp–pine interaction.

## 1. Introduction

Siricidae is a family of economically important wood-boring insects, whose adults and larvae are invasive alien species often intercepted at ports of entry [1]. *Sirex nitobei* (Hymenoptera: Siricidae), which is native to Asia, attacks conifers such as the *Larix* and *Pinus* species. It was first reported in China in 1980 [2]. In 2016, *S. nitobei* was found to damage *Pinus sylvestris* var. *mongolica* in Inner Mongolia, causing the weakening and death of a considerable number of pines [3]. However, the hosts of *S. nitobei* are many and widespread; they include *P. sylvestris* var. *mongolica*, *P. tabuliformis*, *P. armandii*, *P. thunbergia*, and *P. massoniana*. So, *S. nitobei* has expanded from its earliest place of discovery in China to 1750 km southwest, 1450 km northwest, and 2200 km northeast [4]. As a species closely related to invasive woodwasp *S. noctilio*, *S. nitobei* can also be considered a potential high-risk invasive species.

*S. nitobei* females attack pines through their reproductive act of oviposition. The ovipositor consists of a dorsally fused section, the lance, and ventrally of two isolated and parallel sections, the lancets. The sheath consists of two obvious sections, the basal and apical sections separated by a narrow membrane about halfway. The mode of connection structure resembled that of T-branch pipe. Eggs and arthrospores were deposited in the ovaries and mycangia, respectively. And the ovaries, mycangia, and venom reservoir of female were linked together by ovipositor [5]. For a male, it is only responsible for mating, so there is no venom gland and mycangia in its abdomen. Using their ovipositors [6,7], females drill through the cambium of host trees and into the xylem, where they inject their eggs, venom gland secretions, and arthrospores of a symbiotic white-rot fungus, *Amylostereum areolatum* [8,9,10,11]. Importantly, a combination of venom and fungus, after successful establishment, can kill host trees [12,13]. The presence of venom alone can only cause the needles to turn yellow and wither. Increased stem respiration and decreased photosynthate transport in host trees have been attributed as early physiological responses to the venom. Some unknown active principles in the venom are transported rapidly and efficiently from the oviposition site to the needles and cause tissue distortion, desiccation, and the collapse of phloem cells. By these means, woodwasp venom reduces the effectiveness of host resistance mechanisms, thereby promoting the successful establishment of the fungus and enhancing the survival of eggs and larvae [12,14].

As *S. nitobei* continues to expand its range, increased losses to the forest economy are inevitable. Therefore, to prevent host death and economic loss, it has become particularly important to characterize *S. nitobei* venom and identify its protein composition. Recent advances in molecular biology and high-throughput sequencing technology have substantially contributed to the identification of Hymenoptera venom components. A multi-omics approach—the integration of transcriptomics, proteomics, and genomics—has been used for this purpose, providing abundant molecular information about venom components [15,16,17,18,19,20,21,22]. This integrated research approach has also laid the foundation for the identification and development of new biomolecular technologies with agroforestry applications [23].

In recent studies, the combination of transcriptomics and proteomic has been used in Hymenoptera to identify the venom of parasitic wasps, such as the identification of venom from *Pimpla turionellae* (Hymenoptera: Ichneumonidae) and *Bracon nigricans* (Hymenoptera: Braconidae) [24,25]. In contrast, few venom components have been identified from a limited number of woodwasps. As far as we known, only in *S. noctilio*, approximately 90 proteins were predicted by the venomics approach [26].

In this study, we constructed the venom gland transcriptome and venom proteome of *S. nitobei* and combined the two omics using the venomics approach to conduct an in-depth investigation into the composition of *S. nitobei* venom. In accordance with previous studies, our results accurately describe the protein composition of *S. nitobei* venom to better understand its toxic effects in the hosts. This knowledge provides a deeper understanding of the woodwasp–pine interaction and a basis for further studies on the molecular mechanism of how woodwasp venom proteins regulate the defense system of pines. Moreover, it helps in the identification of woodwasp-resistance genes in pines, providing the possibility of cultivating pines resistant to woodwasps.

## 2. Results

### 2.1. Summary and Analysis of Venom Gland Transcriptome Data

A cDNA library for venom glands from *S. nitobei* with three biological replicates was constructed and each were sequenced using HiSeq 4000. A total of 138,974,566 raw reads were generated and 137,607,450 clean reads were acquired after removing the adaptor, short, and low-quality reads (Table 1). The average numbers of GCs of clean reads were 43.40% and the average of Q30 values were 93.87%. All high-quality clean reads were de novo assembled into transcripts using Trinity software. A total of 18,410 transcripts were acquired. After further redundancy treatment of these transcripts, A total of 15,036 unigenes were generated with an average length of 1085 bp and 1857 bp at the N50 level for *S. nitobei* (Figure S1).

From the unigenes, 11,560 encoding proteins that contain ORFs were identified and used for subsequent analysis. Gene annotation was performed using BLASTp search against the UniProt database, and 6400 homologous proteins were found against the Swiss-Prot database (Table S1). We identified 198 unigenes with similar toxin-related proteins and venom proteins among these sequences by using BLASTp to search the Tox-Pro database. Classification of these proteins mainly consisted of “Calglandulin”, “Alpha-latrocrustotoxin-Lt1a (Fragment)”, and “Venom serine protease (Bi-VSP)”. Detailed results and expression levels of these 198 unigenes are shown in Table S2.

**Figure 1:**
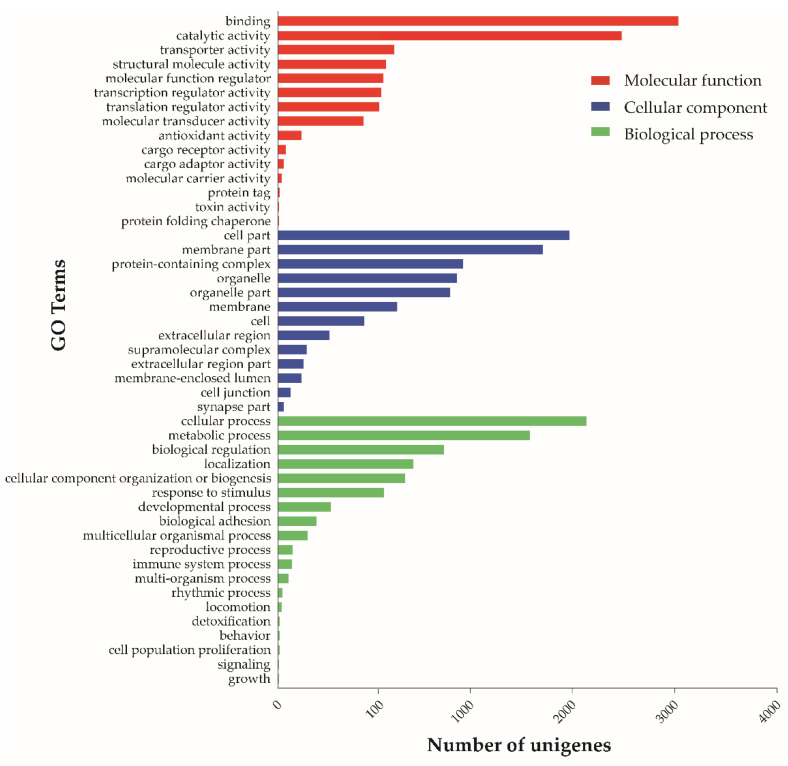
Gene ontology (GO) level 2 functional annotation of the unigenes from *S. nitobei* venom glands at the biological process, cellular component, and molecular function levels.

Blast2GO was used to perform functional annotation of all unigenes. A total of 7771 unigenes (51.68% of the total assembly) were assigned to Gene Ontology (GO) categories. At GO level 2, unigenes were divided into 52 functional groups based on the GO classification results of three main ontological categories: 2946 unigenes at the biological process level, 3401 at the cellular component level, and 4605 at the molecular function level (Figure 1). At the molecular function level, unigenes were further classified into “binding” and “catalytic activity” subcategories (4122 and 3391, respectively). Unigenes in other categories were fewer than 400.

A total of 537 encoding protein sequences with signal peptides at the N-terminus were identified using SignalP v5.0, indicating that these sequences may be secreted proteins with important functions. The expression levels (TPM values) of all unigenes are shown in Table S3, and 1495 unigenes had TPM values > 1. Screened sequences with signal peptides and high expression levels were used for subsequent integrated analysis with the proteome data.

### 2.2. Identification of Venom Proteins by Integrated Transcriptomic and Proteomic Analysis

**Figure 2:**
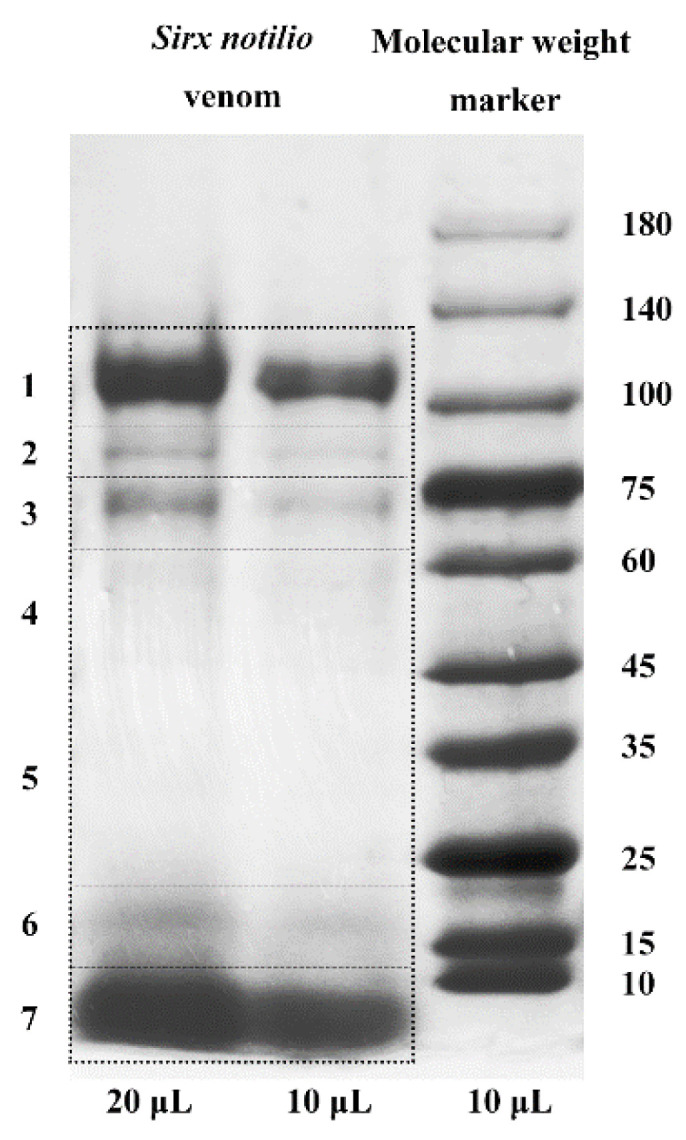
SDS-PAGE of the crude venom extract from *S. nitobei*. Proteins were separated on 4–15% gradient gels and stained with Coomassie brilliant blue R-250. The left two lanes are the venom proteins, and the right lane is the molecular weight marker.

*S. nitobei* venom protein components were separated by SDS-PAGE, and the apparent molecular masses of the separated bands ranged from 5 kDa to 140 kDa. Figure 2 shows that the *S. nitobei* venom protein extract separated into seven protein parts and two major protein bands with a molecular mass ranging between 100–120 kDa (high) and <10 kDa (low). These proteins were analyzed by LC-MS/MS and Table S6 lists the general quantitative information of the identified proteins. We identified 45,842 spectra in total and mapped 1095 proteins in *S. nitobei* VGs (Tables S4 and S7). The proteins predicted from the venom gland transcriptome were used as the reference database to match the resulting peptides. The resulting proteins were used for subsequent analysis with an extensive venom protein set in which the proteins had signal peptides and were highly expressed (TMP > 20). After screening under these conditions, we identified 13 venom proteins that may perform important functions. These proteins contain 2–87 peptides with masses ranging from 9 kDa to 92 kDa. Among these secretory venom proteins, 3 proteins were novel (without annotation information), and 10 proteins showed high sequence similarity with protein sequences in the Swiss-Prot database (Table 2).

### 2.3. Verification of the Expression of the Selected Venom Gland Genes

**Figure 3:**
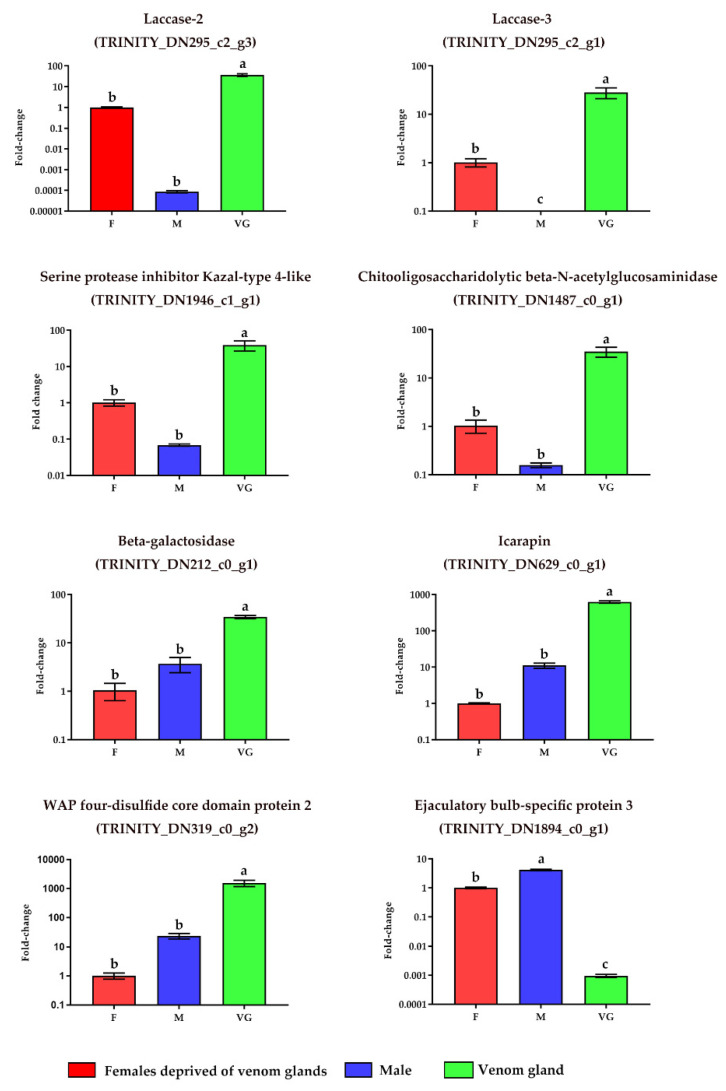
Specific expression of selected genes in the venom gland. Results showing that the abundance of selected genes measured by qRT-PCR in females without venom glands, males, and venom glands. The results are presented as the mean fold changes of three independent biological replicates and the females without venom glands are used as calibrator. Different letters (a, b, c) were denoted that the mean values are significantly different (*p* < 0.05).

We performed qRT-PCR from cDNA synthesized from total RNA extracted, whole adult males, and females without venom glands (i.e., venom glands removed) to verify the effectiveness of the integrated transcriptomic and proteomic analysis to screen and identify venom proteins that play an important role in the venom blend of *S. nitobei*, A total of eight protein-encoding genes with signal peptides at the *N*-terminus were selected. In the venom gland transcriptome, these genes had higher expression levels and were successfully annotated with similar genes in the Swiss-Prot database. RNA extracted from females without venom glands was used as the calibration sample. The results show that in Figure 3, a significantly higher transcription level in venom glands was observed for seven genes considered (Table S8): laccase-2 (*p* < 0.001), laccase-3 (*p* < 0.005), serine protease inhibitor Kazal-type 4-like (*p* < 0.005), chitooligosaccharidolytic beta-*N*-acetylglucosaminidase (*p* < 0.001), beta-galactosidase (*p* < 0.001), icarapin (*p* < 0.001), wap four-disulfide core domain protein 2 (*p* < 0.001). Moreover, compared with venom glands, ejaculatory bulb-specific protein 3 is significantly higher in males and females without venom glands.

## 3. Discussion

Most herbivorous and wood-boring insects cause mechanical damage to plants through feeding and, together with their special elicitors, induce plants to produce a series of physiological and biochemical reactions and to activate defense genes [27,28]. For example, glucose oxidase in *Helicoverpa zea* (Lepidoptera: Noctuidae) saliva and polyphenol oxidase in *Bemisia tabaci* (Hemiptera: Aleyrodidae) saliva are major elicitors of plant defense [29,30]. *S. nitobei* injects venom into the hosts, leading to physiological changes that facilitate the normal growth and development of eggs and larvae in the hosts [12,14,31]. With the continuous improvement and development of bioinformatics methods and next-generation sequencing technology [32], more and more venom components have been identified. This study is the first to construct the transcriptome map of *S. nitobei* VGs. We acquired 15,036 unigenes, of which 8657 annotated unigenes, 6380 unannotated unigenes were aligned. These unannotated unigenes probably consist of short sequences without protein domains or assembly errors, untranslated regions, non-coding RNA, and novel genes, which suggest that there are some uncharacterized sequences in *S. nitobei* venom. All unigenes were functionally annotated by Blast2GO, and the results showed that the most of unigenes were classified into “binding” and “catalytic activity” subcategories. However, the classification of unigenes in the venom gland transcriptome of other wasps is different. Such as *B. nigricans*, the “cell part” and “metabolic process” contained the most unigenes [25]. It is reasonable to assume that they produced specific venom components due to host differences during the evolution process.

The combination of transcriptomics and proteomics was used for the discovery of the elicitors of *S. nitobei* venom involved in host regulation. A large proportion of sequences (57.43% of the total unigenes) were neither annotated in the UniProt nor Swiss-Prot database. Although these proteins are likely to contain some venom components with important functions, it is difficult to identify and verify these proteins from a functional point of view. We identified a robust set of proteins with high expression levels in *S. nitobei* venom glands. These could play an important role in venom production because the expression level is often related to biological functions [33,34]. Among them, the proteins selected for subsequent analysis were based on the criteria of (1) predicting the presence of signal peptides using SignalP v5.0; (2) having a high expression level; and (3) identifying proteins related to parasitoid–host interactions. Moreover, the qRT-PCR results of these genes showed that their expression was significantly higher in *S. nitobei* venom glands than in females without venom glands and males, which further confirmed their specific functions in the venom blend.

Laccase-3 (TRINITY_DN295_c2_g1, TPM = 562.87) identified by transcriptome and proteome analyses contains three cupredoxin domains and was highly expressed in *S. nitobei* venom glands. During the integrated transcriptomic and proteomic analysis, we also found that laccase-2 (TRINITY_DN295_c2_g3, TPM = 3,138.77) was highly expressed. However, it lacked a possible signal peptide at the *N*-terminus as determined using SignalP v5.0. This is likely because the ORF region of the sequence encoding this enzyme is not complete; it has a 5′-terminal deletion. Laccase was discovered in the sap of the Japanese lacquer tree *Rhus vernicifera* [35]. It is noteworthy that laccases isolated from plants, fungi, bacteria, and insects have different sequences and functions [36,37,38]. Plant laccases are involved in the biosynthesis and polymerization of lignin [39,40]. In fungi, laccases are involved not only in the oxidation of phenolic compounds, but also in the oxidation of non-phenolic substrates such as polycyclic aromatic hydrocarbons, synthetic dyes, aromatic amines, and other non-obvious laccase substrates [41,42]. Bacterial laccases are involved in morphogenesis, pigmentation processes, and protection against ultraviolet light and oxidizing agents [43,44]. In termites, two laccases are produced by the salivary glands, secreted into the foregut, and play a role in lignocellulose digestion in the gut [45]. In *S. nitobei*, venom is produced in the venom glands, stored in the venom sac, and is injected into the host plant along with the eggs via the ovipositor. Thus, the two laccases of *S. nitobei* venom play an important role in influencing host physiological changes. However, their specific functions need to be further verified.

A protein (TRINITY_DN1946_c1_g1) with no sequence similarity to the protein sequences in the Swiss-Prot database was identified. However, the results of the protein family prediction using InterProScan showed that this protein characterized the Kazal family (IPR039932) and contained a Kazal-type serine protease inhibitor (KSPI) domain. KSPIs comprise a large family of protease inhibitors [46]. Previous studies of KSPIs mainly focused on vertebrates, particularly mammals and birds [47]. So far, several KSPIs with various functions have been reported in insects. For example, in *Oryctes rhinoceros* (Coleoptera: Scarabaeidae), a single-domain KSPI played an important role in protection against bacterial infection [48]. In *Bombyx mori* (Lepidoptera: Bombycidae), a three-domain KSPI was speculated to resist infection by pathogenic microorganisms [49]. In addition, KSPIs play a key role in the venom of Hymenoptera, especially parasitic wasps. In *Apis cerana* (Hymenoptera: Apidae) venom, a microbial KSPI inhibited subtilisin A and proteinase K [50]. In *Nasonia vitripennis* (Hymenoptera: Pteromalidae), two KSPIs were identified and proved to disrupt the prophenoloxidase activation of the host hemolymph [46]. Therefore, KSPIs perform various functions in insects, particularly those related to insect immune response and host immunity impact. The function of KSPI in the *S. nitobei*–host interaction is worthy of study.

A chitooligosaccharidolytic β-*N*-acetylglucosaminidase (GlcNAcase) (TRINITY_DN1487_c0_g1) showed 67% sequence identity (BLASTp, E-value = 0) with a chitooligosaccharidolytic GlcNAcase of *Orussus abietinus* (Hymenoptera, Orussidae) (GenBank: XP_012277932.1). The enzyme contains a catalytic domain (PF00728) and a β-acetylhexosaminidase-like domain (PF14845). Chitooligosaccharidolytic GlcNAcase was purified from the integument tissues of *B. mori* and *Manduca sexta* (Lepidoptera, Sphigidae), and full-length cDNA was isolated and sequenced, suggesting that GlcNAcases hydrolyze chitooligosaccharides to monomers and recycle them to remodel the exoskeleton during metamorphosis [51,52,53,54]. A chitooligosaccharidolytic GlcNAcase in the venom gland of *Cotesia chilonis* (Hymenoptera: Braconidae) exhibits 57% sequence identity with a homolog found in *N. vitripennis* that is highly expressed specifically in the venom glands [20]. Although the function of GlcNAcase in *S. nitobei* venom was not studied further, it is reasonable to assume that it may participate in the degradation of host tissues.

Beta-galactosidase (TRINITY_DN212_c0_g1) contains a Glyco_hydro_35 (PF1301) domain, which is a member of the glycoside hydrolase 35 family (IPR001944). This β-galactosidase (β-gal) showed 65.10% sequence identity with a β-galactosidase isoform X2 (GenBank: XP_015601851.1) from *Cephus cinctus* (Hymenoptera: Cephidae). β-Gal is abundant in nature and is widely present in animals, plants, and microorganisms. The enzyme can hydrolyze the β-1,3 and β-1,4 glycosidic bonds, meanwhile, it exhibited a high level of transgalactosylation activity in hydrolysis of lactose in milk [55]. In plants, β-gal can change the stability of some cell wall components and degrade or dissolve pectin, a long chain polygalacturonic acid [56,57]. Two β-gal isoenzymes isolated from apple showed strong decomposition ability to arabinogalactan derived from *Larix gmelinii*. During *S. nitobei* oviposition, β-gal may make it easier for the ovipositor to penetrate the xylem by softening the host cell wall.

An icarapin-like protein (TRINITY_DN629_c0_g1) identified by integrated transcriptomic and proteomic analysis, and its encoding sequence with a signal peptide showed 57.62% sequence identity with a homolog found in *C. cinctus*. Icarapin was first identified in the venom of *A. mellifera carnica* (Hymenoptera: Apidae) [58] and has since been identified in various Hymenoptera species, including *A. cerana*, *Megachile rotundata* (Hymenoptera: Megachilidae), *Bombus terrestris* (Hymenoptera: Apidae), *Polistes dominula* (Hymenoptera: Vespidae), and *Solenopsis invicta* (Hymenoptera: Formicidae), as well as in *Leptinotarsa decemlineata* (Coleoptera: Chrysomelidae), *Drosophila grimshawi* (Diptera: Drosophilidae), mosquitoes, termites, flies, and thrips. In these insects, icarapin-like proteins shared a consensus sequence of approximately 40 residues. Interestingly, most of the identified icarapin homologs were predicted based on either transcriptomic or genomic sequences. At the proteomic level, the protein was only identified as a component of the venom in *A. mellifera*, *P. dominula* [59], and *S. noctilio* [26]. Icarapin can be used as a marker allergen for honeybee venom sensitization and can help identify primary sensitization to the venom [60]. However, icarapin contains no functional domains and its function is unknown, which explains why no domains were predicted in *S. nitobei* venom.

Waprin-Thr1-like protein with a conserved four disulfide bond arrangement (PF00095) was identified as a member of the whey acidic protein (WAP) family. The encoding sequence showed 68.09% sequence identity (E-value = 2e^−41^) with a homolog found in *C. cinctus*. A characteristic of the WAP family is that it contains a conserved four disulfide bond arrangement and variable intervening residues [61]. The WAP family has been identified in various tissue types from several organisms and performs various functions, including protease inhibition, antimicrobial activity, and immunomodulation [62,63,64]. However, most studies mainly focused on the function of antimicrobial activity. A snake venom-like waprin identified in the skin secretions of *Ceratophrys calcarata* (Anura: Leptodactylidae) showed potential antimicrobial activities, suggesting that waprin plays a key role in innate immunity [65]. In *Oxyuranus microlepidotus* (Reptilia: Elapidae), waprin can kill microorganisms by membrane disruption; interestingly, it can only do so against some Gram-positive bacteria [66].

Besides venom, female woodwasps inject arthrospores of the symbiotic fungus *A. areolatum* into host trees during oviposition [10,67]. The symbiotic fungus provides essential nutrients for the growth and development of eggs and larvae. It grows slowly and its ability to occupy the niche is weak. Thus, we speculate that the key role of waprin is to inhibit bacterial growth to ensure the smooth colonization of the symbiotic fungus.

## 4. Conclusions

In this study, a combination of transcriptomics and proteomics was used to describe main components of *S. nitobei* venom and contributes to the expansion of the limited information available for the venom of woodwasps that introduce hosts to produce a series of physiological and biochemical reactions, which contributes to the colonization of the symbiotic fungus and the growth and development of eggs and larvae. The most abundant venom components of *S. nitobei*, mainly proteases, are likely involved in the degradation of the host plant lignocellulose and changes in cell wall stability. Enzymes with antimicrobial activity can inhibit other bacteria or fungi to ensure the normal growth of symbiotic bacteria of tree wasp. The identification of these venom components verifies that the infection mode of *S. nitobei* is coordinated damage of “insect-venom-fungus”. Moreover, with the invasion of *S. noctilio* and the continuous expansion of *S. nitobei* range, it is important to study the function of woodwasps venom during the process of infecting host plants. The work carried out in this study will hopefully help researchers to better understand the interaction mechanism between Siricidae insects and host plants.

## 5. Materials and Methods

### 5.1. Sample Collection and Tissue Dissection

Pine (*P. sylvestris* var. *mongolica*) logs infested by *S. nitobei* were collected in April 2017 in Yushu, Jilin Province (China, 44° 50′ 20″ N, 126° 32′ 6″ E). Logs were incubated at room temperature in an insectary of Beijing Forestry University until woodwasps emergence. Female woodwasps individuals were anaesthetized on ice after their emergence and immediately dissected under a microscope (×40) (Leica M205C, Heidelberg, Germany) to isolate the venom sac and venom glands. The collected pairs venom glands were preserved in 1.5 mL microtubes and stored at −80 °C until total RNA extractions.

### 5.2. RNA Extraction

Venom glands of *S. nitobei* with three biological replicates were subjected to RNA extraction. Total RNA was extracted using the RNeasy Plus Mini Kit (Qiagen, Hilden, Germany) following the manufacturer’s protocol. A total of 98.2 μg RNA were obtained from 15 female woodwasps venom glands. DNAse treatment (PureLink^®^DNase, Thermo Fisher Scientific, Massachusetts, USA) was performed to eliminate genomic DNA. RNA purity and concentration were detected using the NanoDrop 2000 (Thermo Fisher Scientific, Waltham, MA, USA). RNA integrity and the RIN values were assessed by agarose gel electrophoresis with the Agilent 2100 Bioanalyzer (Agilent Technologies, Palo Alto, CA, USA).

### 5.3. cDNA Library Construction and Transcriptome Sequencing

mRNA enrichment, fragment interruption, cDNA synthesis, addition of adapters, PCR amplification, and RNA sequencing were performed on three RNA pools at Majorbio Corporation (Shanghai, China). mRNA samples were purified and fragmented using the TruSeq RNA Sample Prep Kit (Illumina, San Diego, CA, USA). Then, total RNA was purified with oligo(dT)-attached magnetic beads to elute poly-(A+) mRNA, followed by fragmentation using a DNA fragmentation kit (Ambien, Austin, TX, USA). Next, under the action of reverse transcriptase, the first-strand cDNA was generated using random-hexamer primers (Invitrogen, Carlsbad, CA, USA) under the following reaction conditions: 25 °C for 10 min, 42 °C for 50 min, 70 °C for 15 min. Further, Second Strand Master Mix and dATP, dGTP, dCTP, and dUTP mix were added for the synthesis of second-strand cDNA (16 °C for 1 h). The purified fragmented cDNA was then incubated at 30 °C for 30 min in the presence of End-Repair Mix, followed by A-tailing. Ligation of adaptors and 15 rounds of PCR master mix were performed to enrich the cDNA fragments. The products were quantified precisely using the TBS-380 (Tuner Biosystems, Sunnyvale, CA, USA), followed by purification using the MinElute Gel Extraction Kit to obtain a cDNA library. The cDNA library was sequenced using Illumina HiSeq 4000 (Illumina, San Diego, CA, USA). The transcriptome raw data have been submitted to NCBI database with the SRA accession number PRJNA718718.

### 5.4. De Novo Assembly and Analysis

To obtain high-quality clean reads for sequence assembly, the adaptor, short (<20 bp), and low-quality sequences were removed from the raw reads. Simultaneously, the percentage of bases with a Phred value > 20 (Q20) and the percentage of bases with a Phred value > 30 (Q30) were calculated. No reference genome database was available for use for *S. nitobei* transcriptome data. De novo transcriptome assembly of clean short reads was performed with the Trinity software package [68] using the default parameters. The transcripts obtained by Trinity were used as reference sequences, and the longest transcript of each gene was considered a unigene for subsequent analysis. The open reading frame (ORF) prediction process provided by TransDecoder tool was used to predict all assembled transcripts, and the protein family (Pfam) database was used to correct the prediction results. The functions of these sequences were annotated based on the Swiss-Prot database (E-value < 10^−5^) using BLASTx, retrieving protein functional annotations with the highest sequence similarity for the direction of these sequences. The signal peptides of these transcripts were predicted using SignalP v5.0. To identify putative toxins, all sequences were aligned against the Tox-Pro database (http://www.uniprot.org/program/Toxins, accessed on 8 June 2021), which has been categorized into toxin and venom proteins [69]. Gene expression levels were estimated in terms of transcripts per million (TPM) values calculated using RNA-Seq by Expectation Maximization (RSEM) v1.3.1 and the Bowtie2 parameter mismatch 0.

### 5.5. Extraction and SDS-PAGE Analysis of S. nitobei Venom Proteins

*S. nitobei* venom sacs were removed from −80 °C refrigeration and transferred to a grinding tube. After adding the protein lysis solution, the tube was shaken three times (40 s each time) in a tissue grinder. The crude extract was lysed on ice for 30 min and then centrifuged at 16,000× *g* at 4 °C for 30 min. The resulting supernatant was the venom protein extract.

About 13 μg of the venom protein extract was mixed with 2× Laemmli buffer and heated at 100 °C for 10 min. *S. nitobei* venom proteins were fractionated by sodium dodecyl sulfate-polyacrylamide gel electrophoresis (SDS-PAGE) on 4%–15% gradient gels (Solarbio, China) with HEPES running buffer. A 10 μL pre-stained protein was used as a molecular weight marker (kDa) of separated *S. nitobei* venom proteins. And we added 20 μL and 10 μL venom protein to the first two lanes of the SDS-PAGE gel, respectively. Electrophoresis was performed at 150 V for 40 min. The resulting gel was stained with Coomassie brilliant blue R-250 (Thermo Fisher Scientific) and stored at 4 °C in 5% acetic acid for further experiments.

### 5.6. LC-MS/MS Analysis and S. nitobei Venom Protein Identification

A 100 μg venom protein sample was dissolved in triethylammonium bicarbonate buffer (TEAB) to a final concentration of 100 mM TEAB. 100 μL of the lysate and 10 mM Tris (2-carboxyethyl) phosphine were mixed for 60 min at 37 °C, and then 40 mM idoacetamide (IAM) was added for 40 min at room temperature in the dark. Pre-cooled acetone was added to the sample (v_sample_:v_acetone_ = 6:1) for 4 h at −20 °C, then centrifuged at 10,000 rpm at 4 °C for 20 min to obtain the precipitate. The precipitate was re-dissolved in 100 μL of 100 mM TEAB and then proteins were digested by adding trypsin in the 1:50 trypsin-to-protein mass ratio overnight at 37 °C. After trypsin digestion, the polypeptide mixtures were collected, desalted, and dried in a vacuum concentrator.

The reverse-phase high pH liquid chromatography (RP-HPLC) separation was achieved on Waters ACQUITY UPLC (Waters, USA) with an C18 column (1.7 μm, 2.1 mm × 150 mm, Waters, Milford, MA, USA) at a flow rate of 200 μL/min. A total of 20 fractions were collected and were subsequently pooled into 10 fractions. The EASY-nLC 1200 (Thermo, USA) with C18 (70 μm × 25 cm, Thermo Fisher, USA) was used for liquid chromatography (LC) analysis at a flow rate of 300 nL/min. The mass spectrometry (MS) was performed by Q-Exactive HF X (Thermo, USA) and the Dynamic Exclusion^TM^ settings were as follows: the 20 most intense precursor ions (m/z ranged from 350 to 1300 Da/e, acquisition mode: DDA) were selected to be scanned. For HSD MS scans with R = 70,000 and MS/MS scans with R = 17,500, the automatic gain control target values were 3 × 10^6^ and 1 × 10^5^, and the maximum injection times were set at 20 ms and 50 ms, respectively. Dynamic exclusion of 18 s was applied to avoid the redundancy of multiple fragmentations for MS/MS spectra of the same ions.

The resulting MS/MS spectra were investigated using PEAKS Studio 8.5 (Bioinformatics Solutions, Waterloo, ON, Canada). The database selected to investigate the proteome data comprised the translated *S. nitobei* transcriptome. Carbamidomethyl was set as static modification, and the oxidation of methionine (M) was set as dynamic modification with two maximum missed cleavage sites. Mass tolerance of the fragments was set at 0.05 Da and that of the precursor ions was set at 10 ppm. The filter parameters of the results were set to peptide false discovery rate (FDR) of 1% and unique peptide ≥1.

### 5.7. Quantitative RT-PCR (qRT-PCR) Analysis

To confirm the result of transcriptomic and proteomic analyses, qRT-PCR was used to assess the expression profiles of selected genes. We extracted total RNA from females without venom glands, males, and venom glands, respectively. The TB Green II PCR Kit was used to measure the differential relative expression of selected genes. All primers were designed using Primer premier 6.0 software (Premier, Quebec, Canada) and showed in Table S5. PCR conditions for all primer sets were as follow: 95 °C for 30 s, 40 cycles of 95 °C for 5 s, 60 °C for 30 s. Elongation was set at 95 °C for 10 s. Melt curve analysis was performed at 60–95, with 0.5 °C increments for 5 s per step. The 60 s ribosomal gene was used as an internal reference. The relative genes expression was calculated using the 2-ΔΔCT methods. The one-way analysis of variance (ANOVA) and Tukey’s test was used to determine whether the expression data held any significant differences at *p* < 0.05. The mean fold change of three independent biological replicates was used for graphical presentation.

## Figures and Tables

**Table 1:** Summary of *S. nitobei* venom glands transcriptome sequencing data.

Sequencing and Assembly Parameters	Value
Total number of raw reads	138,974,566
Total number of clean reads	137,607,450
Average of GC Content (%)	43.4
Average of Q20 (%)	97.54
Average of Q30 (%)	93.87
Total number of transcripts/unigenes	18,410/15,036
Average length of transcripts/unigenes (bp)	1171.30/1085.37
N50 length of transcripts/unigenes (bp)	1982/1857

**Table 2:** Potential venom proteins identified from *S. nitobei* by integrated transcriptomic and proteomic analysis.

Unigene ID	TPM	Best Hit in SwissProt Database	Protein Family	Peptides	Mass
TRINITY_DN185_c0_g2	725.73	No hits found	None	12	10,259
TRINITY_DN295_c2_g1	562.87	No hits found	Cupredoxin	53	71,463
TRINITY_DN1946_c1_g1	342.52	No hits found	Serine protease inhibitor Kazal-type 4-like	2	11,479
TRINITY_DN1487_c0_g1	205.83	Chitooligosaccharidolytic beta-*N*-acetylglucosaminidase P49010 *Bombyx mori*	Beta-hexosaminidase	87	68,318
TRINITY_DN212_c0_g1	140.43	Beta-galactosidase P23780 *Mus musculus*	Glycoside hydrolase, family 35- Beta-galactosidase 1-like	54	72,755
TRINITY_DN2737_c0_g2	42.43	Ferritin subunit P41822 *Aedes aegypti*	Beta-hexosaminidase	7	24,849
TRINITY_DN1504_c0_g2	40.96	Basement membrane-specific heparan sulfate proteoglycan core protein P98160 *Homo sapiens*	None	3	11,856
TRINITY_DN629_c0_g1	33.07	Icarapin Q5EF78 *Apis mellifera carnica*	None	9	23,713
TRINITY_DN319_c0_g2	32.62	WAP four-disulfide core domain protein 2 Q8CHN3 *Rattus norvegicus*	WAP superfamily	4	12,716
TRINITY_DN10373_c0_g1	29.39	Probable salivary secreted peptide D8KY57 *Bombus ignitus*	Transcription activator MBF2	4	12,877
TRINITY_DN1894_c0_g1	24.37	Ejaculatory bulb-specific protein 3 Q9W1C9 *Drosophila melanogaster*	Insect odorant-binding protein A10/Ejaculatory bulb-specific protein 3	13	14,079
TRINITY_DN2893_c1_g2	23.82	Carboxypeptidase Q Q6GQ29 *Xenopus laevis*	Carboxypeptidase Q	34	54,699
TRINITY_DN2064_c0_g2	22.69	Peptidyl-prolyl cis-trans isomerase FKBP14 Q9NWM8 Homo sapiens	None	12	25,268

## Data Availability

The raw sequences have been deposited at SRA-NCBI (Accession Number: PRJNA 718733).

## Supplementary Materials

The following are available online at https://www.mdpi.com/article/10.3390/toxins13080562/s1, Figure S1: The unigene length distribution of the venom glands of *S. nitobei*; Table S1: Characteristics of similarity search of unigenes against Uniprot database. Table S2: Characteristics of similarity search of unigenes against Tox-Pro database; Table S3: Unigenes abundance expressed in TPM; Table S4: Potential venom proteins identified from *S. nitobei* by LC-MS/MS; Table S5: Primers used for qRT-PCR analysis of selected venom components; Table S6: The general quantitative information of the identified proteins by LC-MS/MS; Table S7: Identification information of analysis by LC-MS/MS; Table S8: One-way ANOVA of ΔCt values recorded in: females without venom glands, males and venom glands.
