# Supplementary material for: Proteo-Transcriptomic Characterization of Sirex nitobei (Hymenoptera: Siricidae) Venom
Source: Toxins (Basel). 2021 Aug 11;13(8):562. doi: 10.3390/toxins13080562 (PMC8402507; doi:10.3390/toxins13080562)
Supplement: Supplementary file 1 [file toxins-13-00562-s001.zip › toxins-1324462 supplementary Figure S1 and Tables S5, S7 and S8.pdf]

Supplementary Materials: Proteo-Transcriptomic Characterization of *Sirex nitobei* (Hymenoptera: Siricidae) Venom

Chenglong Gao, Lili Ren, Ming Wang, Zhengtong Wang, Ningning Fu, Huiying Wang, Xiaochen Wang, Tegen Ao, Wensheng Du, Zijin Zheng, Huadong Li and Juan Shi

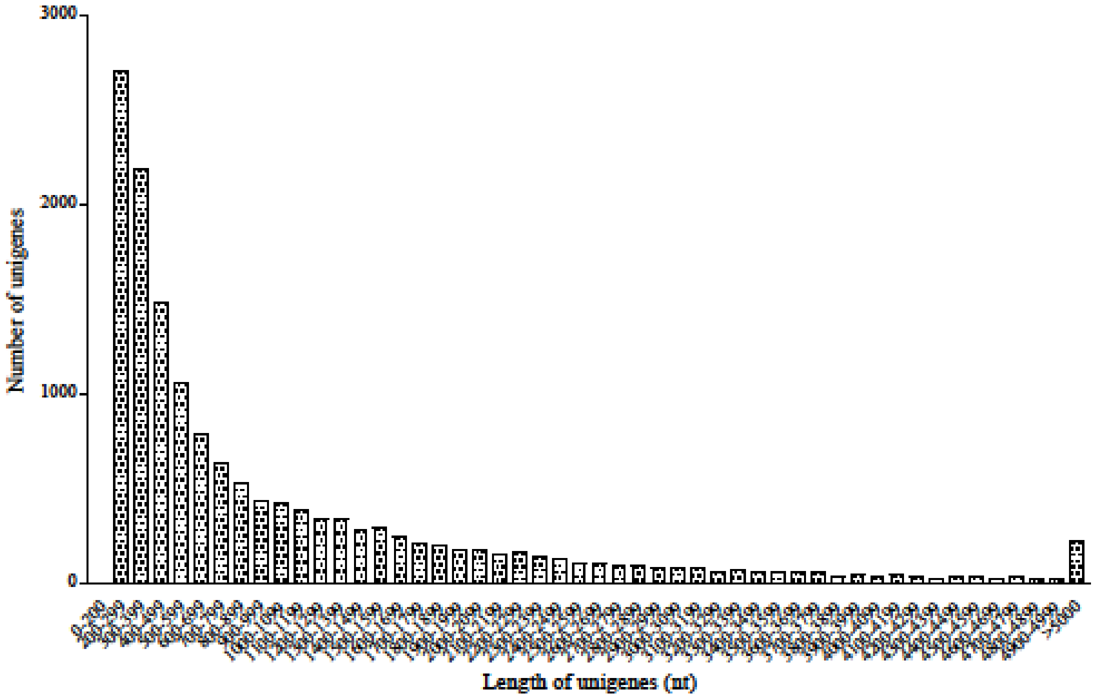

Figure S1. The unigene length distribution of the venom glands of *S. nitobei.*

Table S5. Primers used for qRT-PCR analysis of selected venom components.

Primer	Sequence
Laccase-2	F: ATCCTGGCGATTGGTTGCTTCATT<br>R: TGGCGGTAATGCCGGTACGT
Laccase-3	F: GACGATTCACTGGCACGGCATT<br>R: GGCGTTGGAGTGGTCTCATTGG
Serine protease inhibitor Kazal-type 4-like	F: GCACATCTCAGCATTCCTTCATTCG<br>R: TCTCGTATCCTTGGCGGTTGGT
Chitooligosaccharidolytic beta-*N*-acetylglucosaminidase	F: AGTGTAACTTCGCCACCAAGAACAA<br>R: CCTCTACGGACGCTACCATTGCTA
Beta-galactosidase	F: ACTGTATTGCGGTCCACTGTTAGC<br>R: AAGCCTGCGACACGAAGTATATGG
Icarapin	F: GTGCCATGAACAGACTCAGAGAACA<br>R: TCCGTCACATCCTAAGCAGTCAGAG
WAP four-disulfide core domain protein 2	F: GAACAATCGCCAGTGCATCATCCT<br>R: TGTATCCACCATCGTCTCCGAAGTT
Ejaculatory bulb-specific protein 3	F: TGACCTTCTCACTGCCTGCTCTC<br>R: CGAACGCCTGCTCAAGAACTATGT
60S ribosomal protein<br>(housekeeping gene)	F: TTCGTGTACGGCGTTGTGTTCTT<br>R: TGTTGTACCTGGAGCCATGAGAGT

Table S7. Identification information of analysis by LC-MS/MS.

Total Spectrum	Identified Spectrum	Peptide Number	Protein number
45842	11823	6762	1095

Table S8. One-way ANOVA of ΔCt values recorded in: females without venom glands, males and venom glands.

Transcript	F	Significance
Laccase-2	$F_{2,6}$=156.7	$p < 0.0001$
Serine protease inhibitor Kazal-type 4-like	$F_{2,6}$=30.34	$p < 0.001$
Chitooligosaccharidolytic beta-*N*-acetylglucosaminidase	$F_{2,6}$=53.63	$p < 0.001$
Beta-galactosidase	$F_{2,6}$=352.3	$p < 0.0001$
Icarapin	$F_{2,6}$=485.4	$p < 0.0001$
WAP four-disulfide core domain protein 2	$F_{2,6}$=50.31	$p < 0.001$
Ejaculatory bulb-specific protein 3	$F_{2,6}$=584.7	$p < 0.0001$
